# Annotation and curation of uncharacterized proteins- challenges

**DOI:** 10.3389/fgene.2015.00119

**Published:** 2015-03-31

**Authors:** Johny Ijaq, Mohanalatha Chandrasekharan, Rajdeep Poddar, Neeraja Bethi, Vijayaraghava S. Sundararajan

**Affiliations:** Bioclues Organization, HyderabadIndia

**Keywords:** hypothetical proteins, annotation, functional prediction, protein–protein interactions, drug design research, public repository

## Abstract

Hypothetical proteins (HPs) are the proteins predicted to be expressed from an open reading frame, making a substantial fraction of proteomes in both prokaryotes and eukaryotes. Genome projects have led to the identification of many therapeutic targets, the putative function of the protein, and their interactions. In this review we enlist various methods linking annotation to structural and functional prediction of HPs that assist in the discovery of new structures and functions serving as markers and pharmacological targets for drug designing, discovery, and screening. Further we give an overview of how mass spectrometry as an analytical technique is used to validate protein characterisation. We discuss how microarrays and protein expression profiles help understanding the biological systems through a systems-wide study of proteins and their interactions with other proteins and non-proteinaceous molecules to control complex processes in cells. Finally, we articulate challenges on how next generation sequencing methods have accelerated multiple areas of genomics with special focus on uncharacterized proteins.

## Introduction

Proteins are biological macromolecules translated from DNA to perform myriad functions. As a structural entity, they participate in the regulation of genes to perform function as enzymes or catalysts further playing a role in immune system or as a transporter. With the phenotype of an organism depending on the proteins expressed from the genotype, there are different types and classes of proteins based on their composition, configuration, property, and function. Added to this big diversified world of proteins, a new-fangled race called ‘Hypothetical proteins’ (HPs), involved to describe as functional candidates cannot be ignored. The HPs are proteins that are predicted to be expressed from an open reading frame (ORF), but for which there is no experimental evidence of translation. They constitute a substantial fraction of proteomes in both prokaryotes and eukaryotes with a majority of them included in humans and bacteria (Desler et al., 2012). Many HPs show as ‘hypothetical’ when the genome is just sequenced; this is because of lack of annotation. Comparative genomics shows that a substantial fraction of the genes in sequenced genomes encodes ‘conserved hypothetical proteins’ (CHPs). CHPs are the proteins that are conserved among organisms from several phylogenetic lineages but for which there is no functional validation. Genome sequencing has flooded our information base with novel genes of unpredictable functions. Though genome projects have led to the identification of many therapeutic targets, the putative function of the protein, and their interactions could be predicted for only fewer than half of them. In the recent past, an effort has been made to define CHPs as a large fraction of genes in sequenced genomes encoding phylogenetic lineages but those that have no functional characterization for these ‘therapeutic’ targets (Galperin and Koonin, 2004).

## The Current Status

**FIGURE 1:**
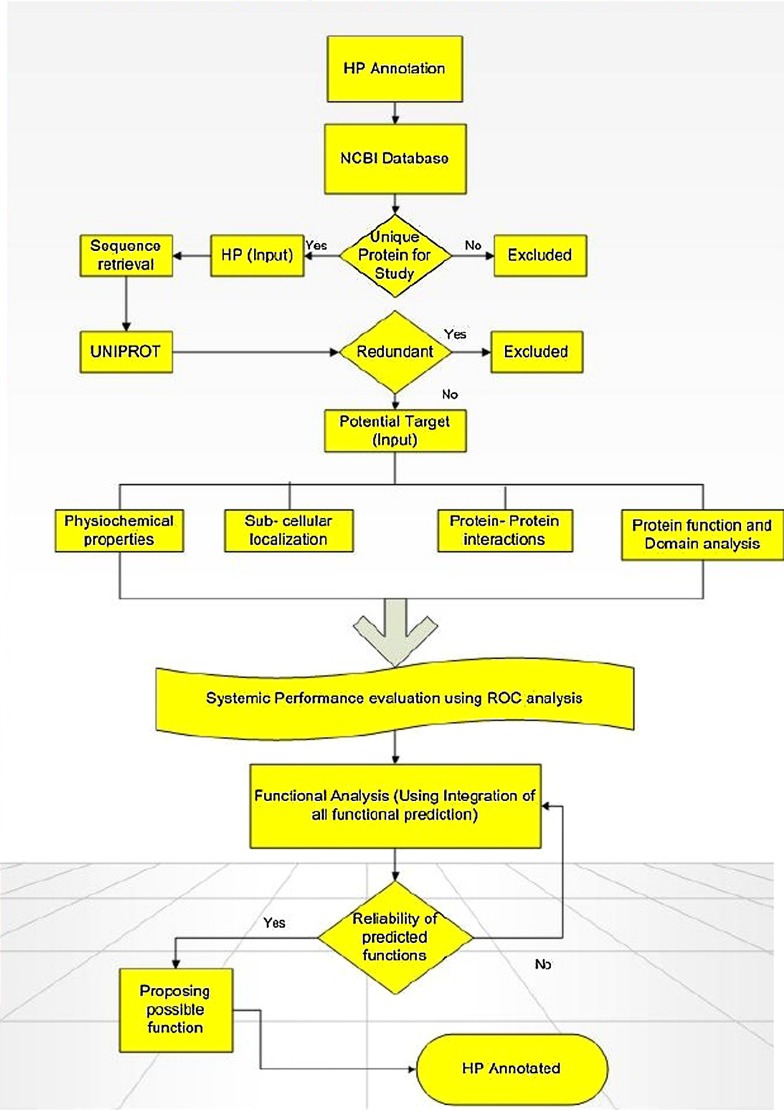
**Flowchart showing the computational framework used for annotating function of hypothetical proteins (HPs). Adapted from Shahbaaz et al. (2013)**.

As on October 08, 2014, the GenBank labels about 48591211 HPs sequences in NCBI on which 7234262 are in eukaryotes and 34064553 are in bacteria. As on date, humans have an approximately 1040 HPs with conserved domains. Come next generation sequencing (NGS), there has been a huge interest in deciphering the function of these HPs not just limited to the sequences generated from traditional sequencing but just to check whether or not any new sequences are generated from NGS. HPs turn up during the genome analysis by bioinformatic tools in the process of identifying new genes. As these tools are pre-fed with instructions for finding the ORF in the genome, they return all possible sequences including those without any protein analog in the protein database or showing less identity to known, annotated protein. There are several *in silico* methods available for the functional predictions of HP, however, no single tool is sufficient enough to perform the annotation all by itself. With fallacy of using several predictors, we firmly reason that using different combination of prediction tools would help reach consensus and validate them to have a significant role which can further be proven by experimental analysis (Sivashankari and Shanmughavel, 2006; Benso et al., 2013; see **Figure 1**).

## Annotation Linked to Structural and Functional Prediction

Annotation of HPs from a particular genome helps in the discovery of new structures and new function which further allows them to be classified into additional protein pathways and cascades. They also serve as markers and pharmacological targets for drug design, discovery, and screening (Shahbaaz et al., 2013). Analyzing and annotating the functions of HPs in pathogenic microorganisms’ causes multiple types of diseases in humans and animals is of utmost important because it would be useful in docking studies for aiding in drug discovery. Furthermore, detection of HPs helps in the discovery of so far unknown or ever predicted genes which would be of great benefit to genomics (Mohan and Venugopal, 2012). Amid several advanced bioinformatics methods developed, we have also incorporated descriptive prediction of proteins with unknown function, viz., homology, database searches for physiochemical properties, subcellular localization, protein classification, domain and motif analysis, protein–protein interactions, etc. (Suravajhala and Sundararajan, 2012). We have reviewed such tools used for functional annotation of HPs in **Table 1**. Recently, a conceptualized outline for ‘Omics Experiments Using Bioinformatics Analogies’ have been designated to represent HPs as an example (Suravajhala and Bizzaro, 2015). The predictions need to be authenticated or validated by *in vitro* and/or *in vivo* experiments to further characterize the predicted functionality. Moreover, *in silico* methods are designed for functional prediction of a protein, but not specifically designed to conform whether or not a protein is hypothetical (see **Figure 1**).

**Table 1:** Methods used for protein characterization and annotation.

**List of bioinformatics tools and databases used for sequence based function annotation**		

**S.no**	**Software**	**Function**

**A**	**Sequence similarity search**	
1	Basic local alignment tool (BLAST)	Used for finding similar sequences in protein databases

**B**	**Physiochemical characterization**	
2	ExPASy -- Protparam tool	Used for computation of various physical and chemical parameters like molecular weight, isoelectric point (Pi), amino acid composition, atomic composition, extinction co-efficient, instability index, aliphatic index, and grand average of hydropathy (GRAVY)

**C**	**Sub-cellular localization**	
3	signalP	Predicts signal peptide cleavage sites.
4	secretomeP	Used for identifying proteins involved in non-classical secretory pathway.
5	PSORT B	Predicts subcellular localization of bacterial proteins.
6	PSLpred	Predicts subcellular localization of proteins from Gram-negative bacteria.
7	CELLO	Assign localization to both prokaryotic and eukaryotic proteins
8	TMHMM	used to authenticate whether the protein is a membrane protein or not.
9	HMMTOP	Predict transmembrane topology.

**D**	**Domain analysis and protein**	
10	Pfam	Collection of multiple protein sequence alignments
11	SVMprot	SVM (Support vector machine based classification of proteins
12	SYSTERS	For grouping of proteins on the basis of their functions.
13	SUPERFAMILY	Hierarchical domain classification of PDB structures. NCBI Entrez protein database search of domain architecture
14	CATH (Class, Architecture, Topology, Homology)	Used for finding protein similarities across evolutionary distances based on domain architecture. Classification based on HMM--HMM search. PANTHER is a
I5	CDART (The conserved domain architecture	comprehensively organized database of protein families and
	retrieval tool)	sub-families, their evolutionary relationships in the form of
		phylogenetic trees
16	PANTHER (Protein analysis through evolutionary relationships)	Identification and annotation of protein domains.
17	SMART	Automatic hierarchical clustering of the protein sequences
18	ProtoNet	

**E**	**Motif Analysis**	
19	InterProScan	Searches interPro for motif discovery. It is the integration of
		several large protein signature databases.
20	MOTIF	used for Motif discovery.
21	MEME suite	Database searching for assigning function to the discovered motifs.

**F**	**Protein--Protein interaction**	
22	STRING	Used for predicting protein--protein interactions.

**List of some wet lab experiments for protein characterization**		

	**Method**	**Function**

**A**	**Chromatographic separations**	
1	Gel filtration chromatography	Separates proteins based on their size (which is closely related to their molecular weight)
2	Ion- exchange chromatography	Purify proteins according to their overall charge
3	Affinity chromatography	Separates proteins based on their affinity to bind to a known ligand.

**B**	**Electrophoresis**	
4	SDS-PAGE	Separates protein according to molecular weight and allows the measurement of the molecular weight in comparison with marker proteins.
5	Isoelectric focusing	Separates proteins based on their PI on a polyacryl-amide gel with a PH gradient.
6	2D-Electrophoresis	Isoelectric focussing is often used in conjunction with SDS-PAGE to give a very powerful method of protein characterization by separating the sample of protein first by isoelectric point and then by molecular weight.

**C**	**Spectroscopic analysis**	
7	NMR spectroscopy	For determining three dimensional structure of proteins
8	Mass spectrometry	For protein identification and characterization.

**D**	**Others**	
9	Yeast two hybrid assay	For studying protein--protein interactions.
10	Phage display method	For studying protein--protein interactions
11	Microarray analysis	For systems-oriented study of proteins
12	Next generation sequencing	For high-throughput sequencing of genome and proteome analysis.

## Wet- Lab Experiments are Used to Confirm the Candidate Hypothetical Protein

Although gene prediction programs using various bioinformatics tools have become more accurate and sensitive, analysis of HPs, there is a want of more reliable evidence for existence and function of predicted proteins (Shin et al., 2004). Identifying HPs starts with cell culture and sample fractionation, i.e., fair separation of protein mixture (Lubec et al., 2005). Once the sample is prepared it is subjected to two dimensional electrophoresis (2-DE) and mass spectrometric analysis. Two- dimensional gel electrophoresis (2-DGE) with immobilized pH gradients (IPGs) combined with identification and characterisation of resolved proteins by mass spectrometry (MS) is currently the core technology for proteomics. Both are essential for studying protein expression, activity, regulation, and modifications at cellular level (Shin et al., 2004). 2-DE is routinely applied for separation and parallel quantitative expression profiling of large sets of complex protein mixtures such as whole cell lysates. 2-DE separates complex mixtures of proteins according to the differences in their isoelectric point (p*I*), molecular mass (*M*_r_), solubility, and relative abundance. In addition, it produces a map of intact proteins (proteome map), which helps in studying the changes in protein expression level, isoforms, or post-translational modifications, thus providing the global view of proteins expressed in any cell or tissue type. The 2-DGE is highly efficient with respect to reproducibility, handling, resolution, and separation of very acidic and/or basic proteins. Depending on the gel size and pH gradient used, 2-DGEs can resolve more than 5000 proteins simultaneously, and can detect and quantify <1 ng of protein (Gorg et al., 2004).

While biochemical characterization of proteins provide insight of gene function, physiochemical properties of the protein such as molecular weight, stability, proper folding, etc. have to be determined. Conventional technologies of protein separation and characterization such as chromatographic separation, protein and DNA electrophoresis, cell sorting, affinity assays (e.g., immunoassays), spectroscopic analysis have been miniatured by microfluidic technologies. Technologies such as microfluidics and other *lab-on-a-chip methods* rely on assays that are rapid and inexpensive (Whitesides, 2006). Microfluidics provides a powerful platform to study protein–protein interactions that play a major role in assigning the putative function to the HPs. As most of the genome-wide functional annotations are based on *in silico* methods, studying protein–protein interactions on a proteome scale can give experimental evidence to the functional annotation and concomitantly can fill the gaps left by *in silico* methods. Recently developed Microfluidics large scale integration (mLSI) technology integrates 1000s of micromechanical values thus replacing conventional automatic methods of genomic and proteomic analysis and further enabling 100s of assays to be performed in parallel with multiple reagents (Melin and Quake, 2007; Meier et al., 2013).

Mass spectrometry is a powerful analytical technique for validating protein coding genes. It analyses and quantifies 1000s of proteins from complex samples and thus permits the characterisation of putative gene products at the level of translation (Tanner et al., 2007). MS provides high-throughput analysis of two-dimensional gels that are used for separation of complex mixture of proteins. Proteins resolved by 2-DGE are identified and analyzed by MS (You and Wang, 2007). Matrix-assisted laser desorption ionization–mass spectrometry (MALDI–MS) is an efficient analytical method for large-scale identification of proteins (Fountoulakis and Langen, 1997). It identifies a protein by matching molecular masses of peptide fragments derived from total proteome digests with all fragment masses from a database of known protein (Henzel et al., 1993). This technique of identifying the proteins by matching their experimentally obtained masses to the theoretical peptide masses generated from a protein database is known as peptide mass mapping or peptide mass fingerprinting technique (Thiede et al., 2005). The mass spectrum is unique for a specific protein and can be viewed as a collection of fragment masses from a single peptide, known as a ‘mass fingerprint’ (Fountoulakis and Langen, 1997; Marvin et al., 2003). In organisms with small genomes such as microorganisms and yeast, peptide mass fingerprinting has been shown to be very successful for characterisation of proteins, where only matching as few as three to four peptides is enough to identify a protein. For larger genomes as the number of expressed proteins increases, greater identification strategy is required, and is achieved through Tandem MS (MS–MS) approaches. Tandem MS also helps in resolving any ambiguity arising from peptide mass fingerprinting. Recent advancements in MS are introduction of robotic technology to increase sample throughput in a “hands off” manner and using nanospray ionization source to analyze very small sample volumes (nl; Molloy and Witzmann, 2002). Due to its high mass range, high sensitivity, and relative tolerance to common buffer components, MALDI–MS has become a popular method for analysis and characterisation of proteins (Henzel et al., 1993).

## Microarrays and Protein Expression Profiles

Current technologies limit our analysis to only one or two of the parameters to be studied and to only fraction of proteins. Systems-oriented proteomics provide us with integrated understanding of biological systems by studying many components simultaneously. Furthermore it helps us to understand how proteins interact with other proteins and non-proteinaceous molecules to control complex processes in cells and tissues and even whole organism. In systems-oriented proteomics the subset of proteins to be analyzed is well defined such that sequences or collection of proteins are related by function. Microarray technology is well suited to systems-oriented studies. Two features that make microarray technology so well suited to systems-oriented proteomics are 1000s of proteins can be interrogated simultaneously by spotting them on a single slide or similar support and similar proteins can be probed repeatedly with many different molecules under many different conditions by fabricating 100s–1000s of copies of an array in parallel (MacBeath, 2002). Protein microarrays can be used to detect stable protein–protein interactions, transient attractions between enzymes and their substrate, and also interaction of proteins with non-proteinaceous molecules like nucleic acids, lipids, and other small organic molecules. Two types of protein microarrays are defined, protein function arrays and protein-detecting arrays. In protein function arrays 1000s of naïve proteins are immobilized in a defined pattern and can be utilized for massively parallel testing of protein function. The other type, protein-detecting array consist of large numbers of arrayed protein-binding agents and will allow for protein expression profiling to be done at the protein level (Kodadek, 2001). However, even microarrays are established tools for genome and protein analysis, requirement of prior knowledge of the genomic features, cross hybridization between similar sequences, high signal to noise ratios, more requirement of sample (in micrograms), and dependence on PCR-based amplification are some of the limitations with microarrays. This has brought paradigm shift in genomic and proteomic analysis toward NGS-based approaches (Hurd and Nelson, 2009).

Next generation sequencing technologies are way ahead of microarrays and fundamentally altered the genomics research. Experiments that were not technically feasible or affordable previously are now made possible with the advent of NGS technology thus accelerating multiple areas of genomics research. Thanks to many NGS platforms that are available sharing a common technological feature of massively parallel sequencing of clonally amplified or single DNA molecules that are spatially separated in a flow cell (Voelkerding et al., 2009). The NGS has offered rapid and inexpensive sequencing capacity. The high throughput capacity of NGS has enabled us to sequence entire genomes (from microbes to humans), targeted genome sequencing, transcriptome sequencing (RNA-Seq), sequencing of ancient DNA samples, and substantially widened the scope of metagenomic analysis including human microbiome. Chromatin immune-precipitation technique (ChIP) is used to study the DNA–protein interactions in order to understand the role of proteins in gene expression regulation. Combining this technology with NGS platforms has enhanced our understanding of gene expression based cellular responses (Mardis, 2008).The most profound impact of NGS technology has been on the discovery of novel non-coding RNAs (ncRNAs) belonging to distinct classes like miRNAs, siRNAs, snRNAs, snoRNAs, piRNAs, piwiRNAs (Axtell et al., 2007; Brennecke et al., 2007; Houwing et al., 2007; Zhao et al., 2007). Discovery of ncRNA systems in different organisms belonging to diverse set of species is a breakthrough in biological research in recent years as their characterization has enhanced the annotation of sequenced genomes (Mardis, 2008).They play an important role in gene regulation and traditionally as the study of cancer has focused on protein coding genes, these ncRNAs are providing new insights into cancer research (Espinosa and Slack, 2006).

## Conclusion

A comprehensive identification of the HPs is needed for the functional interpretation of fully sequenced genomes and further understanding of the diverse functions of its unique structures, which in turn facilitates search for potential proteins of interest for researchers. Development of computational approaches and programs on elucidation of the functions of CHPs create an opportunity for biologists to produce a complete record of their biological functions and the genes involved. Protein science on the other hand have taken a new look with advances in the chemical synthesis of peptides and site-directed mutagenesis as standard research tools. This creates way for the construction of new proteins with customized structural and functional properties. However, the most important step in this process understands the complex folding patterns of these synthetic polypeptides to form a functional protein. We have tabulated and discussed several *in silico* methods available for the functional predictions of HP from sequence to structural levels like homology search, identification of domains and motifs, comparative analysis, phylogenetic profiling, and so on. Interpreting the physiological function of the HPs could establish greater interest in understanding evolutionary relationship of genes and organisms and would as well assist in drug discovery. We believe with the increase in the amount of sequence data with respect to HPs, there is a pressing need to organize this data and network their function to the existing known sequences. This process would allow us to identify HPs localized to different organelles involved with crucial prime functions, linked to various diseases. A permutation and combination of bioinformatics methods followed by wet lab experiments as listed in the figure above would be very useful for rapid functional annotation of novel proteins and will be useful for design of novel peptides and will have immediate impact on drug design research. Though few databases exist for analyzing HPs, a large public repository exclusive for HPs for ready reference to biologists and researchers around the world, would bring a greater impact and solution to many on-going projects.

## Conflict of Interest Statement

The authors declare that the research was conducted in the absence of any commercial or financial relationships that could be construed as a potential conflict of interest.
